# Improved *de novo* Assembly of the Achlorophyllous Orchid *Gastrodia elata*

**DOI:** 10.3389/fgene.2020.580568

**Published:** 2020-11-19

**Authors:** Shanshan Chen, Xiao Wang, Yangzi Wang, Guanghui Zhang, Wanling Song, Xiao Dong, Michael L. Arnold, Wen Wang, Jianhua Miao, Wei Chen, Yang Dong

**Affiliations:** ^1^School of Life Sciences, Zhengzhou University, Zhengzhou, China; ^2^BGI College, Zhengzhou University, Zhengzhou, China; ^3^State Key Laboratory of Genetic Resources and Evolution, Kunming Institute of Zoology, Chinese Academy of Sciences, Kunming, China; ^4^Jiaxing Synbiolab Biotechnology Co., Ltd., Jiaxing, China; ^5^College of Biological Big Data, Yunnan Agricultural University, Kunming, China; ^6^The Key Laboratory of Medicinal Plant Biology of Yunnan Province, National and Local Joint Engineering Research Center on Germplasm Innovation and Utilization of Chinese Medicinal Materials in Southwest China, Yunnan Agricultural University, Kunming, China; ^7^Department of Genetics, University of Georgia, Athens, GA, United States; ^8^State Key Laboratory of Genetic Resources and Evolution, Kunming Institute of Zoology, Chinese Academy of Sciences, Kunming, China; ^9^Center for Ecological and Environmental Sciences, Northwestern Polytechnical University, Xi’an, China; ^10^Center for Excellence in Animal Evolution and Genetics, Chinese Academy of Sciences, Kunming, China; ^11^Guangxi Key Laboratory of Medicinal Resources Protection and Genetic Improvement, Guangxi Botanical Garden of Medicinal Plants, Nanning, China; ^12^State Key Laboratory of Conservation and Utilization of Bio-Resources in Yunnan, Yunnan Agricultural University, Kunming, China; ^13^College of Agronomy and Biotechnology, Yunnan Agricultural University, Kunming, China; ^14^Yunnan Research Institute for Local Plateau Agriculture and Industry, Kunming, China

**Keywords:** *Gastrodia elata*, genome, achlorophyllous, relaxed selection, non-photosynthetic

## Abstract

Achlorophyllous plants are full mycoheterotrophic plants with no chlorophyll and they obtain their nutrients from soil fungi. *Gastrodia elata* is a perennial, achlorophyllous orchid that displays distinctive evolutionary strategy of adaptation to the non-photosynthetic lifestyle. Here in this study, the genome of *G. elata* was assembled to 1.12 Gb with a contig N50 size of 110 kb and a scaffold N50 size of 1.64 Mb so that it helped unveil the genetic basics of those adaptive changes. Based on the genomic data, key genes related to photosynthesis, leaf development, and plastid division pathways were found to be lost or under relaxed selection during the course of evolution. Thus, the genome sequence of *G. elata* provides a good resource for future investigations of the evolution of orchids and other achlorophyllous plants.

## Introduction

In the autotroph-dominant plant world, the symbiotic relationship between plants and fungi plays an indispensable role in the maintenance of ecosystem (Read, 1991). In an extreme situation, some plants (at least 50 independent origins) have become solely dependent upon their fungal associates for energy source and other nutrients during the course of evolution (Merckx and Freudenstein, 2010). These fully mycoheterotrophic plants often lack a functional photosynthetic mechanism, so they are termed as “non-photosynthetic plants (Merckx et al., 2009).”

The loss of photosynthesis was independently evolved over 40 times in a diverse range of plant families and genera (Bidartondo, 2005). As an evolutionary adaptation to a low-light undergrowth environment, this extreme phenotype was also associated with some quite remarkable parallel evolutionary traits in plant, such as smaller biomass, specialized leaves without stomata, lack of root hair, reduced vascular tissues, and miniatured seeds (Leake, 1994). Even though multiple studies of plastomes from heterotrophic plants have showed reduction in plastome sizes and housekeeping gene numbers (Krause, 2008; Barrett and Davis, 2012; Wicke et al., 2013, 2014), the nuclear genomic basis of the other parallel traits in non-photosynthetic plants remains elusive.

All members of the Orchidaceae family (up to 26,567 species in 880 genera) rely on fungal associates in some or all stages of their life cycle (Leake, 1994; Cai et al., 2015). In particular, more than 200 orchid species from a myriad genera are fully mycoheterotrophic and non-photosynthetic (Leake, 1994; Merckx et al., 2009; Merckx and Freudenstein, 2010). Since *Gastrodia* is one of the largest genera consisting of fully mycoheterotrophic species from a wide geographic area (Cribb and Killmann, 2010), members in this genus are excellent models to investigate the reconfigured traits in non-photosynthetic plants.

**FIGURE 1:**
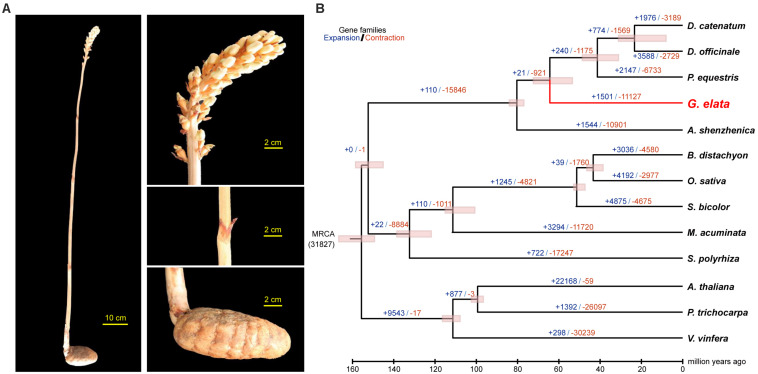
Divergence time and morphology of *G. elata*. **(A)** Morphology of *G. elata*: photos showing a whole plant, flower, stem with degraded blade and tuber. **(B)** Divergence times are indicated by pink bars, expanded gene family numbers by dark blue letters and contracted gene families by orange letters.

In China, *Gastrodia elata* is cultivated for medicinal uses. It is a perennial, non-photosynthetic orchid with an enlarged rhizome and vestigial leaves on an upright flower-bearing stem (Figure 1A). The completion of its life cycle requires at least two types of fungi: *Armillaria* and *Mycena* (Lan et al., 1994; Ning et al., 2010) (Supplementary Figure 1). In this study, we presented a high quality *de novo* assembly of the *G. elata* nuclear genome, which had a higher coverage, contig N50 size, and Benchmarking Universal Single-Copy Ortholog (BUSCO) completeness than that of a previous report (Yuan et al., 2018). Compared with the genomes of photosynthetic orchid species, such as *Phalaenopsis equestris* (Cai et al., 2015), *Apostasia Shenzhenica* (Zhang et al., 2017), and *Dendrobium officinale* (Yan et al., 2015), the new *G. elata* nuclear genome and plastome assemblies showed key gene loss and relaxed selection related to its non-functional photosystem and retarded leaf development. These data demonstrated that the improved *G. elata* genome provides more insights into the genomic and evolutionary mechanisms underlying the morphological and physiological adaptations associated with a non-photosynthetic lifestyle.

## Materials and Methods

### Plant Material, Genomic DNA Extraction, and Library Construction

A flowering *G. elata* Bl. f. glauca S. Chow plant was collected from Zhaotong, Yunnan Province, China (103°43′E, 27°20′N). Genomic DNA was extracted from fresh stems by the Qiagen DNeasy Plant Mini Kit (Cat. No. 69104). After genomic DNA purification and quality validation, 10 paired-end and mate-pair DNA libraries with insert sizes of 169 bp (×2), 300 bp (×2), 374 bp, 545 bp, 753 kb, 2 kb, 5 kb, and 10 kb were constructed using library construction kits (Illumina) as previously described (Liang et al., 2015; Supplementary Table 1).

### Genome Sequencing and Assembly

A whole-genome shotgun strategy was adopted to sequence and assemble the genome of *G. elata*. All ten DNA libraries were sequenced on an Illumina HiSeq 2000 platform and 483.07 Gb of data was obtained. The genome size of *G. elata* was estimated according to the 17-mer frequency distribution with the formula: Genome size = *K*-mer_num/Peak_depth. Total of 84.67 Gb data was retained for 17-mer analysis. The 17-mer frequency distribution plot shows the peak at 66 (Supplementary Figure 2), and the total *K*-mer count is 90,917,019,718, then the genome size is estimated as 1.378 Gb.

The assembly of *G. elata* genome was carried out using the SOAPdenovo. A *de Bruijn* graph was constructed using the parameter “−K 83 −d 3”, then the graph was simplified using “−M 3” parameters to clip tips, remove low coverage links, merge bubbles, and solve tiny repeats. Those repeats on the simplified graph were broken at the boundaries and unambiguous DNA fragments were produced as contigs. Filtered sequencing data was then realigned to contigs with the parameters “−k 83”. Parameter “−F” was used to deal with the paired-end information, and unique contigs were joined to construct scaffolds. Gapcloser (v1.12) was used to fill the gaps between scaffolds with the default parameters.^1^ Contigs short than 100 bp were excluded within the whole assembly process.

The completeness and accuracy of the gap closed assembly was assessed using short insert-sized sequencing reads, conserved genes, and RNA-seq data. Filtered reads from three small-insert libraries (169, 374, and 753 bp) were mapped to the assembled genome using the bowtie2 (version 2.2.9^2^) (Langmead and Salzberg, 2012) with default parameters to estimate the quality of the assembly. The overall mapping rates of these small-insert size reads is higher than 92%, suggesting that most of the *G. elata* genome has been assembled. BUSCOs approach (Waterhouse et al., 2017) was also used to evaluate the completeness of the *G. elata* genome. Three hundred and three (303) conserved single-copy orthologs has been used as reference, of which 248 (81.85%) were identified in our genome assembly (Table 1). Over 74.59 millions of cleaned RNA-seq reads from three different *G. elata* tissues (flower, stem, and tuber) were mapped to the assembled genome with the default settings using the TopHat2 (version 2.0.14^3^) (Trapnell et al., 2009; Supplementary Table 2), and we observed the average mapping ratio of 83%, further validate the high completeness of *G. elata* genome assembly.

**TABLE 1:** Various assembly parameters of the *G. elata* genome.

		*G. elata*
	*G. elata* (This study)	(Yuan et al., 2018)
Sequencing platform	Illumina Hiseq 2000	Illumina Hiseq 2500
Sequenced data (Gb)	483.07	179.1
Genome coverage (×)	350.56	151.78
Estimated genome size (Gb)	1.37	1.18
Assembled genome (Gb)	1.12	1.06
Contig N50 (Kb)	110.03	68.97
Scaffold N50 (Mb)	1.64	4.91
TE proportion (%)	69.81%	66.18%
Total BUSCO groups searched	303	956
Complete BUSCOs	81.85%	67.15%
Duplicated BUSCOs	17.49%	9.21%
Fragmented BUSCOs	3.96%	4.39%
Missing BUSCOs	14.19%	28.45%

### RNA-Sequencing Data Analysis

Overall, 82.37 million transcriptome raw reads were obtained from *G. elata* leaf, stem, and tuber tissues (Supplementary Table 2). First, all RNA-sequencing (RNA-seq) data were filtered by the strict quality control process, then about 74.59 millions cleaned reads were *de novo* assembled using trinity^4^ (Grabherr et al., 2011) with default settings to yield transcripts that prepared for the genome annotation. Next, all RNA-seq reads were mapped to the *G. elata* genome assembly using the TopHat2 (version 2.0.14; see text footnote 3) (Trapnell et al., 2009) with default settings. The fragments per kilobase of exon model per million reads mapped (FPKM) of each predicted protein-coding gene was calculated by the Cufflinks^5^ using default parameters. FPKM > = 0.05 was set as the threshold to identify expressed genes.

### Repeat Sequence Annotation

RepeatMasker (version 3.2.6) with the Repbase TE library was used to identify known transposable elements (TEs) with the default parameters. The library was constructed by generating the consensus sequence of each TE family, which was used for the RepeatMasker to identify additional high and medium copy repeats in the *G. elata* genome assembly. TRF with parameters set to “Match = 2, Mismatch = 7, Delta = 7, PM = 80, PI = 10, Minscore = 50, and MaxPeriod = 2000” was used to predict tandem repeats.

### Protein-Coding Gene Annotation

A combination of *de novo*-, transcriptome-based prediction, and homology aligning were used to process gene annotation. Gene sets from ten species (*Arabidopsis thaliana*, *Brachypodium distachyon*, *Oryza sativa*, *Sorghum bicolor*, *Solanum tuberosum*, *Triticum aestivum*, *Hordeum vulgare*, *P. equestris*, *Dendrobium catenatum*, and *A. shenzhenica*) were used for homology-based predictions, one species at a time. We used the TBLASTN to search the non-redundant protein sequences of each gene set with an E-value < 1e-2. Only regions with homologous blocks longer than 80% of the query protein were retained. The best hits were selected. Then, the EVM was used to construct the gene structures. After repeat sequences were masked using the homology-based approach, three softwares, AUGUSTUS (Stanke et al., 2006), SNAP (Korf, 2004), and GlimmerHMM (Majoros et al., 2004), were used for the genes *de novo* prediction. Information obtained from the homology-based predictions and *de novo* predictions were then integrated in the GLEAN to generate a consensus gene set. Finally, RNA-seq data from a single *G. elata* plant’s flower, stem, and tuber tissues were obtained and assembled for the facilitating of the protein-coding gene annotation.

### Functional Annotation

Best hits were selected from alignments to the SwissProt and TrEMBL databases to assign gene function information. Gene motifs and domains were identified using the InterProScan5^6^ (Jones et al., 2014) by alignment to databases including the ProDom, PRINTS, Pfam, SMART, PANTHER, and PROSITE. According to the corresponding SwissProt and TrEMBL entries information, Gene Ontology (GO) terms and ID for each gene was obtained. Kyoto Encyclopedia of Genes and Genomes (KEGG) protein database was used as reference for gene alignments to obtain KEGG IDs as well as the corresponding pathways information.

### Gene Family Clustering

All proteins from selected 13 species (*Gastodia elata*, *P. equestris*, *D. officinale*, *D. catenatum*, *A. shenzhenica*, *B. distachyon*, *O. sativa*, *S. bicolor*, *Spirodela polyrhiza*, *Musa acuminata, A. thaliana*, *Populus trichocarpa*, and *Vitis vinifera*) were aligned using the BLASTP, then, the gene families were defined using the OrthoMCL (Li et al., 2003). CAFÉ (De Bie et al., 2006) was then used to identify gene family expansions and contractions in *G. elata.* To identify gene family clusters in these species and *G. elata*, we performed all-versus-all protein alignments using the BLASTP with the E-value threshold set to “1e-5.” We used the OrthoMCL to process high scoring segment pairs. The MCL module from OrthoMCL was then used to define final paralogous and orthologous genes with the parameter set as “−abc –I = 1.5”.

### Phylogenetic Tree Construction and Divergence Time Estimation

Those single-copy orthologs identified from gene family cluster analysis of the aforementioned species were used to construct a phylogenetic tree. MUSCLE version 3.6^7^ (Edgar, 2004) was used with default settings to perform multiple sequence alignments. Fourfold degenerate sites of genes were collected and concatenated into a “super sequence” for each species. We used the MrBayes^8^ (Huelsenbeck and Ronquist, 2001) to reconstruct phylogenetic trees between species. The “MCMCTREE” module from the PAML package^9^ (Yang, 2007) was used to estimate the divergence time among species.

### Chloroplast Genome (Plastome) Assembly and Annotation

The clean reads were aligned to the plastomes of *P. equestris* and *D. officinale* (Jheng et al., 2012; Yang et al., 2016) using the bowtie2 with default settings, respectively. Reads extracted from the “paired-aligned” alignments were merged and assembled using the SOAPdenovo with default parameters. Contigs shorter than 100 bp were excluded. Filtered contigs were joined to scaffolds based on the paired-end information and gaps between scaffolds were closured. DOGMA^10^ (Wyman et al., 2004) was used to annotate the protein-coding genes and tRNA genes with the cut-off set to 80%. The boundaries of protein-coding genes and plastome structures were manually checked by comparison to the plastomes of *P. equestris* and *D. officinale*. The linear plot of *G. elata* plastome was yielded by the OGDRAW^11^ (Lohse et al., 2013), followed with some manual adjustments.

### Relaxation Selection Analyses and Symbiotic Gene Analysis

These nuclear-encoded photosynthesis-related proteins were identified based on the National Center for Biotechnology Information (NCBI) database. Genome sequences of seven species (*G. elata, A. shenzhenica, P. equestris, D. catenatum, D. officinale, O. sativa*, and *A. thaliana*) were searched for all of the known plant nuclear-encoded photosynthesis-related genes. Only genes that were in a one-to-one orthologs for every pair of genomes of the seven species were used in our analyses. For genes that have more than one transcript, we aligned all of the possible transcript pairs to all seven species and retained those that provided the highest alignment scores. Alignments and consensus trees were used for posterior molecular evolutionary analysis. We used a gene-level approach based on the ratio of non-synonymous (*K*_*a*_) to synonymous (*K*_*s*_) substitutions rate (ω = *K*_*a*_/*K*_*s*_) to identify potential relaxation of selective constraints, using the CODEML likelihood ratio tests (LRTs) algorithm from the PAML package. First, we tested branch models M0, the simplest model, which has a single ω ratio for the entire tree. Subsequently, we used two-ratio models that allow a background ω ratio and a different ω on the branch of interest. For null hypotheses, we used the one-ratio model, two-ratio model, and more models with a fixed ω = 1 on the branch under analysis. The level of significance for these LRTs was calculated using a χ^2^ approximation, where twice the difference of log likelihood between the models (2ΔlnL) would be asymptotic to a χ^2^ distribution, with the number of degrees of freedom corresponding to the difference in the number of parameters between the nested models.

These Gastrodia antifungal protein (GAFP) were downloaded from the NCBI database. Genome sequences of seven species (*G. elata, A. shenzhenica, P. equestris, D. catenatum, D. officinale, O. sativa*, and *A. thaliana*) were searched for all of the known GAFP genes with blast software. Finally, the genes with E-value ≤ 1e-6 in the comparison result were selected as a candidate GAFP gene. And the protein domain of carotenoid cleavage dioxygenases (CCDs) was downloaded from the pfam website. The hmmersearch software were used for sequence alignment to identify CCDs genes in other species. The identified genes are calculated by *K*_*a*_/*K*_*s*_ to verify whether positive selection occurred in *G. elata*.

### High Performance Liquid Chromatography Analysis of Photosynthetic Pigments

From each sample of *G. elata*’s vestigial scalelike leaves and *P. equestris*’s and *D. Officinale*’s normal leaves, about 1 g of leave tissue was collected and dried as powder. Then, the dried powder was dealt with 100% 40 mL of methanol for half an hour and sonicated for an hour, and then, methanol diluted to 50 mL. The methanol extract was then filtered by the 0.45 μm membrane filter. Ten microliters of filtrate was prepared for high performance liquid chromatography (HPLC). Quantitative analysis of the photosynthetic pigments of three species was performed using the chromatographic column Inertsil ODS-3 (250 mm × 4.6 mm, 5 μm) and the column temperature was maintained at 25°C with the flow rate set as 1 mL × min^–1^. The mobile phase was consisted of A: acetonitrile and 0.05 mol/L of Tris–HC1 buffer (70:3); and B: methanol and n-hexane (5:1). Gradient elution was then used with the following system: 100% A at initiation, 100% A at 18 min, 100% B at 20.5 min, and 100% B at 46 min. 445 nm was used to detect photosynthetic pigments.

## Results

### De novo Assembly of the *G. elata* Genome

The genome size of *G. elata* was estimated to be 1.378 Gb using the *K*-mer distribution analysis (Supplementary Figure 2). All 483.07 Gb clean data (350.56 × genome coverage) were *de novo* assembled into 69,353 contigs (1.11 Gb in total length) with a contig N50 size of 110.03 kb, and 45,884 scaffolds (1.12 Gb in total length) with a scaffold N50 size of 1.64 Mb (Supplementary Table 3). As a result of high genome coverage, our *G. elata* assembly had a much longer contig N50 size (110.03 kb) than that from a previous study (68.9 kb, Table 1; Yuan et al., 2018). Preliminary evaluation of the quality of our assembly showed that at least 92% of the clean reads from different insert-sizes of paired-end libraries could be mapped back to the assembled genome (Supplementary Table 4). In addition, over 82.37 million RNA sequencing reads from each of the flower, stem, and tuber tissues were generated to further verify the quality of the assembly. The overall mapping ratios of these reads to the genome assembly were 90.7% for the flower, 93.8% for the stem, and 67.1% for the tuber (Supplementary Table 2), indicating the high quality of the *G. elata* genome.

The completeness of the genic regions and other genomic elements in our *G. elata* genome was evaluated by the BUSCOs approach (Waterhouse et al., 2017). The result showed that 248 out of 303 (81.85%) near-universal single-copy orthologs were identified in our *G. elata* genome. This number was much higher than that reported in the previous study (67.15% BUSCO completeness, Table 1; Yuan et al., 2018). Additionally, 12 BUSCO genes (3.96%) had fragmented matches, and 43 BUSCO genes (14.19%) were missing in our assembly. Both parameters were lower than that reported in the previous study (4.39 and 28.45%, respectively, Table 1; Yuan et al., 2018). These data demonstrated that our *G. elata* genome had a much higher completeness for subsequent functional analysis.

### *Gastrodia elata* Genome Repeat Analysis

Repetitive DNA elements constituted approximately 68.34% of the acquired genome (Supplementary Table 5). This proportion was higher than those of the other orchids genomes, including *P. equestris* (62%) (Cai et al., 2015), *D. officinale* (63.33%) (Liang et al., 2015), and *A. shenzhenica* (42.05%) (Zhang et al., 2017). Long interspersed nuclear elements (LINEs) and DNA transposons constituted 6.20 and 7.49% of the total genome assembly, respectively (Supplementary Table 5). The long terminal repeats (LTRs) were the most abundant TEs, which constituted 59.95% of the genome (Supplementary Table 5). Notably, Gypsy-type (37.41%) and Copia-type (8.07%) TEs accounted for most of the LTRs (Supplementary Table 6). Sequence divergence line plot of Copia and Gypsy types of TEs shows that both of the two types experienced two recently expansion events during the same periods (Supplementary Figure 3a). Compared to the conserved single copy genes, those duplicated genes (“accessory genes”) in *G. elata* are located closer to the TEs (Fisher’s exact test, *P* < 0.005), suggesting their contribution in gene copy number variation and the important role in *G. elata* evolution (Supplementary Figure 3b).

### Orthologous Gene Analysis in the *G. elata* Genome

Overall, we identified 24,484 protein-coding genes (Supplementary Table 7) with about 10.05 kb average length in the *G. elata* genome that belonged to 11,065 unique gene families (Supplementary Table 8). Besides, we also identified a myriad of microRNAs (1,125), transfer RNAs (1,123), ribosomal RNAs (1,596), and small nuclear RNAs (868; Supplementary Table 9) in the *G. elata* genome.

Phylogenetic analysis with divergence time estimation showed that the *G. elata* diverged from other orchids somewhere between 72.3 and 55.8 million years ago (Mya) (Figure 1B). During the course of evolution, it is apparent that 19 gene families were significantly expanded (*P* < 0.001; Supplementary Table 10), whereas six gene families were significantly contracted in the *G. elata* genome (*P* < 0.001; Supplementary Table 11). KEGG enrichment analyses showed that gene families involved in flavonoid biosynthesis, plant-pathogen interaction, and circadian rhythm were significantly contracted in the *G. elata* genome (Supplementary Table 12). In comparison, the expanded gene families are mainly involved in the glycan, sphingolipid, and galactose metabolisms, and intriguingly, also plant-pathogen interaction (Supplementary Table 13). Since some members of the plant-pathogen interaction gene families were contracted and other members were expanded, it is possible that *G. elata* may have rewired its pathogen resistance pathway when adapting to the low-light environment. Indeed, *G. elata* as a fully mycoheterotrophic plant not only needs to obtain nutrients from the fungi associates, but also have to prevent the fungi from invading into the inner most section of the tuber (Zhang, 1980).

### *Gastrodia elata* Plastome Assembly and Relaxed Selection of Plastid Genes

**FIGURE 2:**
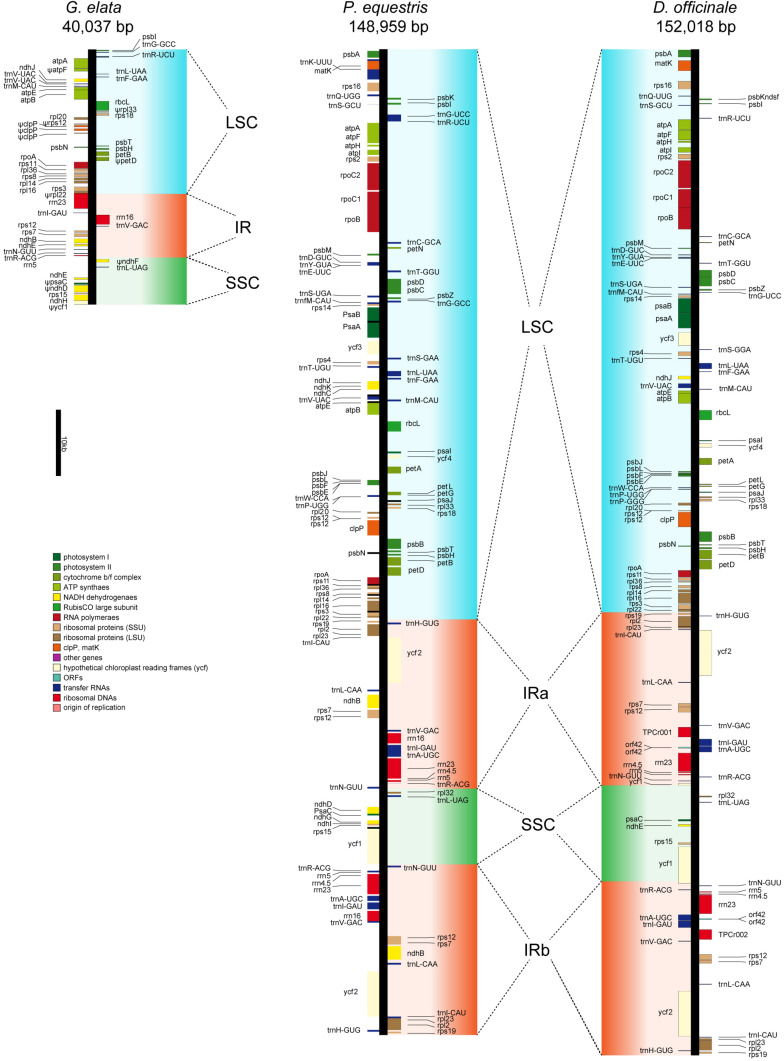
Plastid map of three orchid plants. The plastid of *G. elata* shows significant degradation comparing with *P. equestris* and *D. officinale*. Ψ indicates the pseudogenes.

The conserved plastome structure of most land plants generally is a quadripartite single circular molecule of 100–220 kb. It consists of a small and a large single-copy regions (SSC and LSC) which are separated by a pair of inverted repeats (IRs) (Sandelius and Aronsson, 2009). The plastome of the photosynthetic plant harbors around 100 essential genes that primarily encode RNAs and proteins involved in photosynthesis, transcription, and translation (Daniell et al., 2016). *P. equestris* and *D. officinale* are two photosynthetic orchids with fully functional plastomes as other land plants (Jheng et al., 2012; Yang et al., 2016). The plastomes of these two orchids (with sizes of 148,959 and 152,018 bp, respectively) both have the typical quadripartite single circular molecule structure with relatively complete photosynthesis-related gene sets (Figure 2). In comparison, the plastome of *G. elata* was assembled with the size of 40,037 bp, meaning that more than two thirds of the plastome sequence was lost after the transition to a low-light heterotrophic lifestyle (Figure 2). IRs are considered to play roles in plastome stabilization (Palmer and Thompson, 1982). In the *G. elata* plastome, one copy of IR was completely lost, and the remaining IR sequence was less than half the size of its counterparts in the other two orchids. The *G. elata* LSC retained less than a quarter of the sequence size compared to the two orchid plastomes, and the *G. elata* SSC also lost half of its size.

Only 50 genes were identified in the *G. elata* plastome, among which 10 genes were manually checked to be pseudogenes (with frameshifts or premature stop codons) (Supplementary Table 14). The photosynthesis-related genes in the *G. elata* plastome were carefully checked. The result showed that the paralogs of *psa* and *psb* (photosystem I and II genes), *pet* (cytochrome b/f complex genes), *rbc* (rubisco subunit genes), and *atp* (ATP synthase genes) were completely lost or were found to have incomplete gene structures due to a reduced LSC. Two of the six remaining *ndh* (NADH dehydrogenase) genes were found to be non-functional. All these changes concluded that the genomic basis of photosynthesis in the *G. elata* plastome was highly degenerated. These observations are in accordance with a previous report (Yuan et al., 2018), which includes small assembled plastome sizes (40,026 vs. 35,326 bp), extensively loss of DNA fragments, missing of one copy of IR structure, and loss or non-functional of photosynthesis-related genes.

Natural selection is essential in the maintenance of trait for the plant population. The weakening or disappear of selection strength that leads to trait variations is referred to as “relaxed selection” (Lahti et al., 2009). In its evolutionary history, *G. elata* experienced the transition to a low-light, undergrowth environment (Bidartondo, 2005) and a fully heterotrophic lifestyle. This transition made photosynthesis dispensable for *G. elata* to survive. We therefore hypothesized that the remaining photosynthesis-related plastome genes might be under relaxed selection. Indeed, the *K*_*a*_/*K*_*s*_ values of these genes (includes *psa*, *psb*, *atp*, *pet* genes, and so on) in *G. elata* were higher than *D. officinale* and *P. equestris*, and non-orchid plant species (Supplementary Table 15). This indicates the reduction of functional constraints, and, thus, these genes were under a significantly different selective regime in *G. elata* than in those photosynthetically active species.

### Relaxation of Selective Constraints of Nuclear-Coded Photosynthesis-Related Genes in *G. elata*

**FIGURE 3:**
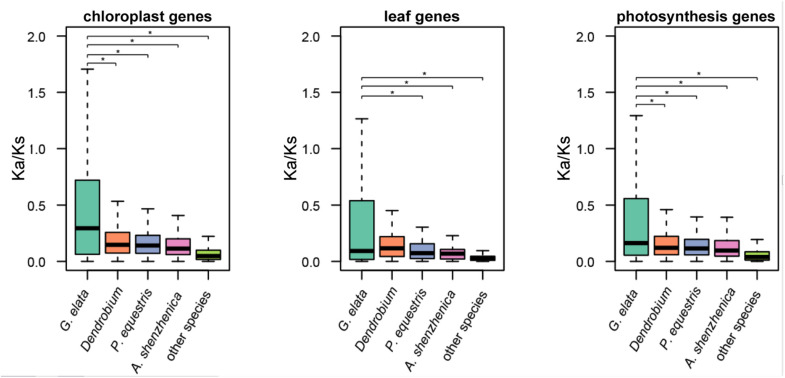
Relaxation of selective constraints on nuclear-encoded genes. *K*_*a*_/*K*_*s*_ ratios of nuclear-encoded plastid genes and genes involved in leaf development in *G. elata*, other orchids (including *D. officinale*, *P. equestris*, *D. catenatum*, and *A. shenzhenica*) and other green plants (including *A. thaliana* and *O. sativa*). *P < 0.05.

We define “photosynthesis-related genes” as all known genes that are involved in the structure, development, and normal function of the plastid and leaf in land plants. The plastome only contains a small part of these photosynthesis-related genes, and the rest are within the plant nuclear genome. Based on the aforementioned hypothesis, we also checked the molecular evolution of the photosynthesis-related nuclear genes in the *G. elata* genome. In brief, they consisted of 4,818 plastid-related genes, 203 leaf development-related genes, and 1,408 genes that were directly involved in the photosynthesis. The *K*_*a*_/*K*_*s*_ values of these genes in *G. elata* were significantly higher than those in other orchids and non-orchid plant species, indicating relaxation of selective constraints on the leaf development and the photosynthesis process (*t*-test and two-way ANOVA test, *p* < 0.05, Figure 3 and Supplementary Figure 4). We also found that the *K*_*a*_/*K*_*s*_ values of these searched genes in orchids were higher than those in non-orchid species. It suggested that there might be a partial relaxation of selection on plastid and leave functions in the photosynthetic orchids due to their symbiotic relationships with fungi. These findings also suggested a positive correlation between the degree of heterotrophy in plants and the non-synonymous mutation rates in genes that were involved in the photosynthetic process, plastid and leave functions.

### Analysis of Symbiotic Genes in *G. elata* Genome

Since *G. elata* cannot perform photosynthesis, it relies on symbiotic genes for nutrition, so symbiotic genes are also attracting attention in the *Analysis of Symbiotic Genes in Gastrodia Genome*. *G. elata* exists underground without leaves and chloroplasts, with the nutrients necessary for growth being supplied by the symbiotic fungus *Armillaria mellea* (Sa et al., 2003) fungus. Gastrodia antifungal protein (GAFP, also known as gastrodianin) was first purified from the cortex of the terminal corm of *G. elata* (Hu et al., 1988). It is mainly distributed throughout the epidermis and cortex layer of *G. elata* (Liu et al., 1993; Hu and Huang, 1994) and shows a strong fungistatic activity against a broad spectrum of fungi, including *A. mellea* (Hu and Huang, 1994), *Rhizoctonia solani*, *Valsa ambiens*, *Gibberella zeae*, *Ganoderma lucidum*, and *Botrytis cinerea in vitro* to reach plants symbiosis with microorganisms (Wang et al., 2001). In the *G. elata* genome, we identified 20 GAFP genes, 10 GAFP genes in *A. shenzhenica*, and 0 in *A. thaliana*, which indicates that GAFP has expanded in *G. elata* (Supplementary Table 16). It is known that strigolactone can stimulate hyphal branching and development of arbuscular mycorrhizal fungi, which increases the chances of an encounter with a host plant (Kretzschmar et al., 2012). Strigolactone is an important signal for the establishment of the symbiosis relationship between *G. elata* and *A. mellea*. Its mechanism of action is similar to that of promoting symbiosis between plants and mycorrhiza. In this article, we have identified the key genes for the biosynthesis of strigolactone CCDs (Delaux et al., 2012). In the *G. elata* genome, 12 CCDs genes were identified, whereas ten and nine CCDs genes were identified in *A. shenzhenica* and *A. thaliana*, respectively. In order to verify whether the CCDs gene has positive selection in *G. elata*, we further performed the *K*_*a*_/*K*_*s*_ analysis. The *K*_*a*_/*K*_*s*_ values of these genes in *G. elata* were significantly higher than those in *D. catenatum* and *P. equestris*. It showed that, compared with some orchid and non-orchid plant species, CCDs have expanded in the *G. elata* genome.

### Genomic Basis for the Achlorophyllous Phenotype in *G. elata*

We next investigated nuclear-encoded genes involved in the plant pigment synthesis in the *G. elata* genome. We detected no copies of four core genes in the chlorophyll biosynthetic pathway (*UROS*, *CAO*, *LPOR*, and *VDE*; Supplementary Figure 5a). This is in line with the result that no signals of major photosynthetic pigments could be detected via the HPLC in the *G. elata* plant tissue (compared to the other two orchids *D. officinale* and *P. equestris*) (Supplementary Figure 5b). Moreover, three essential genes (*PDV1*, *PDV2*, and *ARC3*) involved in the chloroplast division were also not detected (Supplementary Figure 6). These results clearly showed the genomic basis for the achlorophyllous phenotype in *G. elata*.

## Conclusion

The *G. elata* genome provides an illuminating model for probing evolutionary molecular changes associated with leaf loss, plastid degeneration, and plant-fungal symbioses. Both plastid-encoded and nuclear-encoded photosynthesis-related genes in *G. elata* showed evidence of the relaxation of selective constraints. Besides the profound changes in the photosynthetic system, it is possible that future research could identify the underlying mechanisms for the rewiring of basic metabolic pathways, the evolution of TEs, gene death, and generation of new genes, since an improved *G. elata* genome is now available. Thus, the genome sequence of *G. elata* would be a valuable resource for future investigations of the evolution of orchids and non-photosynthetic plants at both the genomic and the whole-organism levels.

## Data Availability Statement

The datasets presented in this study can be found in online repositories. The names of the repository/repositories and accession number(s) can be found below: NCBI database under BioProject PRJNA394702.

## Author Contributions

SSC, GHZ, and WLS collected the samples and performed the experiments. XW, YZW, and XD completed the data analysis. YD, WC, WW, MA, and JHM edited and modified the manuscript. All authors read and approved the manuscript.

## Conflict of Interest

XW was employed by the company Jiaxing Synbiolab Biotechnology Co., Ltd. and she declared that Jiaxing Synbiolab Biotechnology Co., Ltd. plays no role in the funding, design, analysis, and publication of this manuscript. The remaining authors declare that the research was conducted in the absence of any commercial or financial relationships that could be construed as a potential conflict of interest.

## Footnotes

1 http://sourceforge.net/projects/soapdenovo2/files/GapCloser/

2 https://sourceforge.net/projects/bowtie-bio/files/bowtie2/2.3.4.3

3 http://ccb.jhu.edu/software/tophat/index.shtml

4 https://github.com/trinityrnaseq/trinityrnaseq/releases

5 http://cole-trapnell-lab.github.io/cufflinks/cuffdiff/index.html

6 ftp://ftp.ebi.ac.uk/pub/software/unix/iprscan/5/5.32-71.0/interproscan-5.32-71.0-64-bit.tar.gz

7 http://www.drive5.com/muscle/downloads.htm

8 http://nbisweden.github.io/MrBayes/download.html

9 http://evomics.org/resources/software/molecular-evolution-software/paml/

10 http://dogma.ccbb.utexas.edu

11 https://chlorobox.mpimp-golm.mpg.de/OGDraw-Downloads.html
