# Supplementary material for: Improved de novo Assembly of the Achlorophyllous Orchid Gastrodia elata
Source: Front Genet. 2020 Nov 19;11:580568. doi: 10.3389/fgene.2020.580568 (PMC7711107; doi:10.3389/fgene.2020.580568)
Supplement: Supplementary file 1 [file Data_Sheet_1.PDF]

Supplemental Information for:

Improved *de novo* assembly of the achlorophyllous orchid *Gastrodia elata*

Shanshan Chen[1,2,†], Xiao Wang[3,4, †],Yangzi Wang[5, †], Guanghui Zhang[6], Wanling Song[5], Xiao Dong[5], Michael L. Arnold[7], Wen Wang[8,9,10], Jianhua Miao[11,*], Wei Chen[12,13,14*], Yang Dong[5,11,12,14,*]

[1]BGI College, Zhengzhou University, Zhengzhou 450000, China.

[2]Henan Institute of Medical and Pharmaceutical Sciences, Zhengzhou University, Zhengzhou 450000, China.

[3]State Key Laboratory of Genetic Resources and Evolution, Kunming Institute of Zoology, Chinese Academy of Sciences, Kunming 650223, China.

[4]Jiaxing Synbiolab Biotechnology Co., Ltd., Jiaxing 314006, China.

[5]College of Biological Big Data, Yunnan Agricultural University, Kunming 650201, China.

[6]The Key Laboratory of Medicinal Plant Biology of Yunnan Province, National and Local Joint Engineering Research Center on Germplasm Innovation and Utilization of Chinese Medicinal Materials in Southwest China, Yunnan Agricultural University, Kunming, China.

[7]Department of Genetics, University of Georgia, Athens, GA 30602, USA.

[8]State Key Laboratory of Genetic Resources and Evolution, Kunming Institute of Zoology, Chinese Academy of Sciences, Kunming 650223, China.

[9]Center for Ecological and Environmental Sciences, Northwestern Polytechnical University, Xi'an 710072, China.

[10]Center for Excellence in Animal Evolution and Genetics, Chinese Academy of Sciences, Kunming 650223, China.

[11]Guangxi Key Laboratory of Medicinal Resources Protection and Genetic Improvement, Guangxi Botanical Garden of Medicinal Plants, Nanning 530023, China.

[12]State Key Laboratory of Conservation and Utilization of Bio-Resources in Yunnan, Yunnan Agricultural University, Kunming 650201, China.

[13]College of Agronomy and Biotechnology, Yunnan Agricultural University, Kunming, 650201, China.

[14]Yunnan Research Institute for Local Plateau Agriculture and Industry, Kunming 650201, China.

[†]These authors contributed equally to this work.

*These authors are corresponding co-authors.

Corresponding Authors
Jianhua Miao: mjh1962@vip.163.com, (+86) 186-7719-1399
Wei Chen: wchenntr@gmail.com, (+86) 182-9185-2361
Yang Dong: loyalyang@163.com, (+86) 135-7701-6903

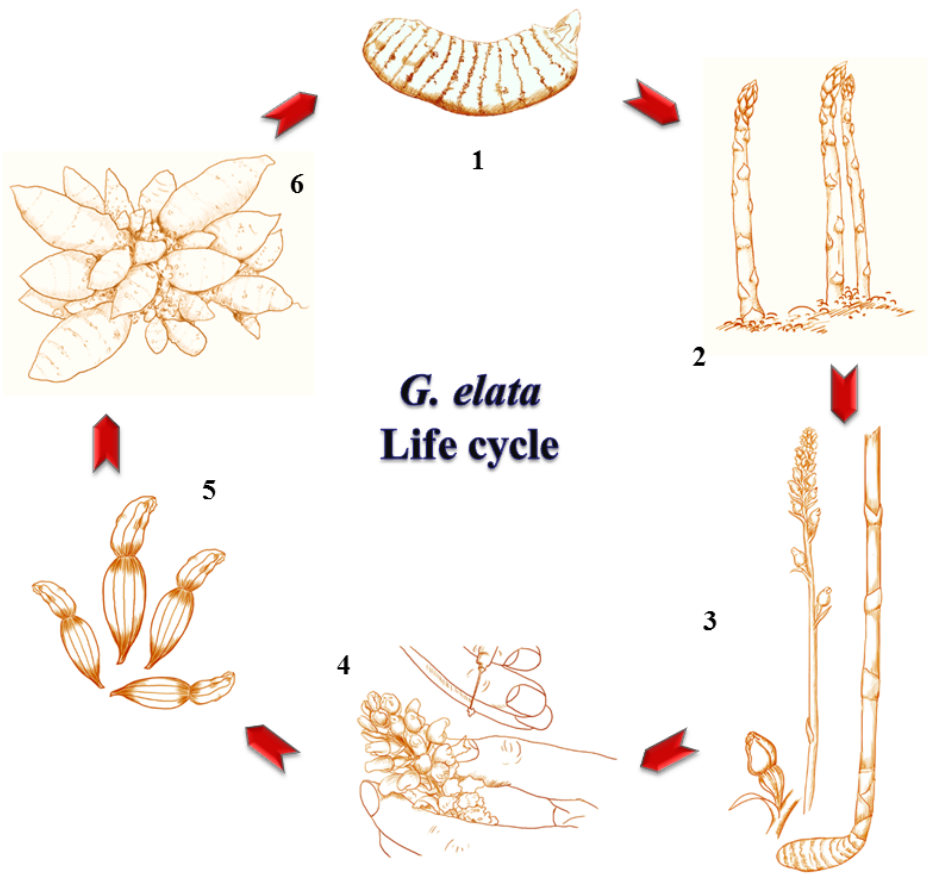

Supplementary Figure 1: The life cycle of *G. elata*.
1: Ripe tuber; 2: Budding stage; 3: Blooming; 4: Artificial pollination; 5: Capsule discloses, seeds germination with germinating fungus; 6: Seed stem with the rhizomorph of *Amillariella mellea.*

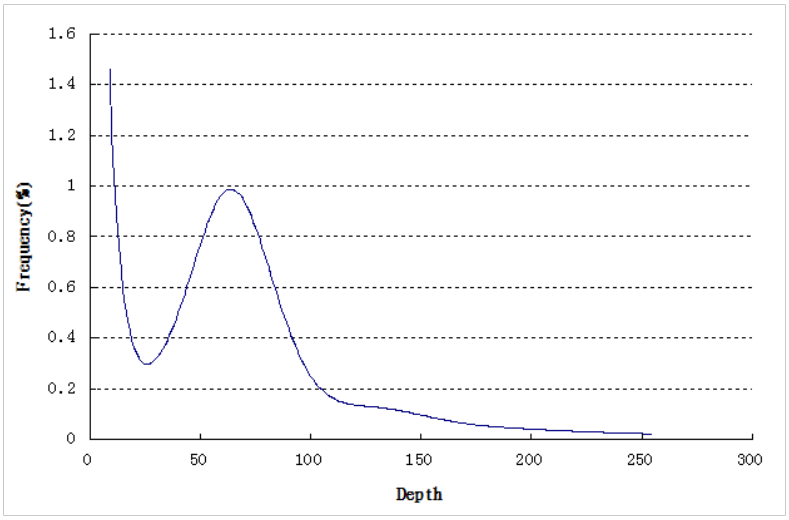

Supplementary Figure 2: 17-Kmer depth distribution.

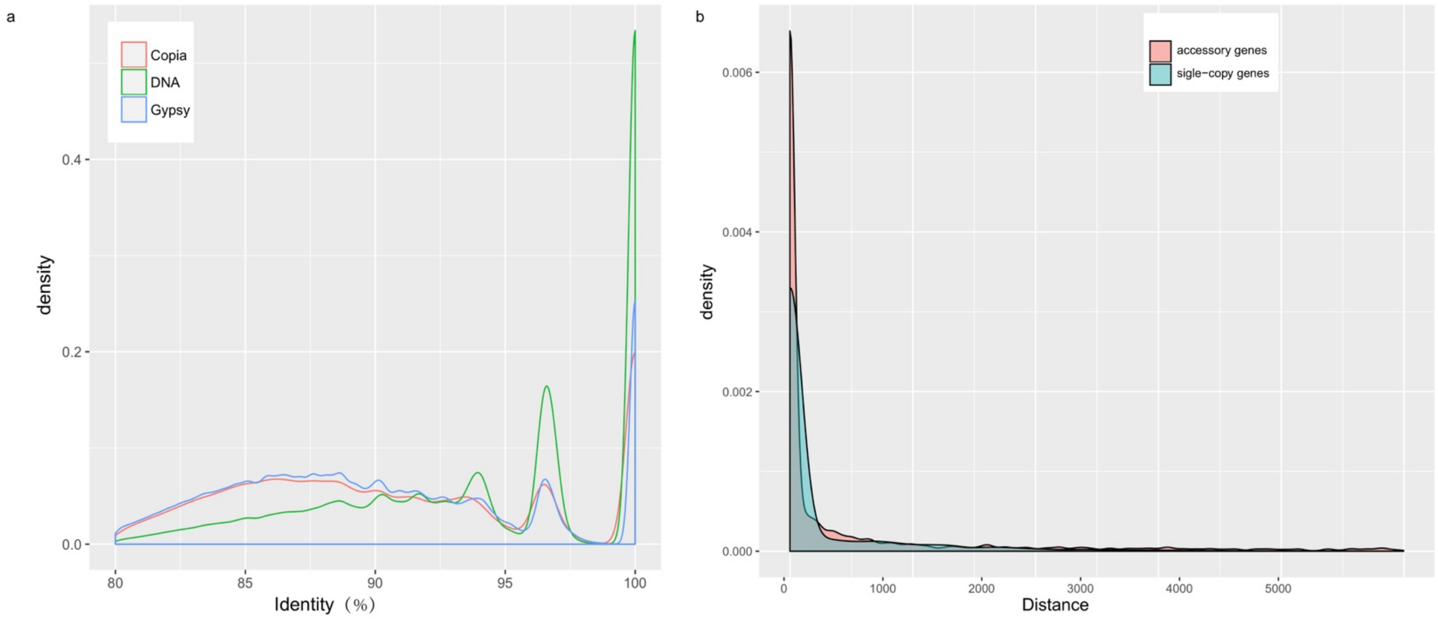

Supplementary Figure 3: Potential impact of transposable elements (TEs) on *G. elata* evolution.

a) Frequency distribution of pairwise sequence identities between copies of Copia- and Gypsy-type LTR retrotransposons and DNA transposons, and their cognate consensus sequences. The similarity between young repeats is higher than those of ancient repeats. b) Distance to the closest TE for the set of *G. elata* single-copy genes (blue) and the set of *G. elata* accessory genes (red). TE-proximal accessory genes are more frequent than TE-proximal single-copy genes.

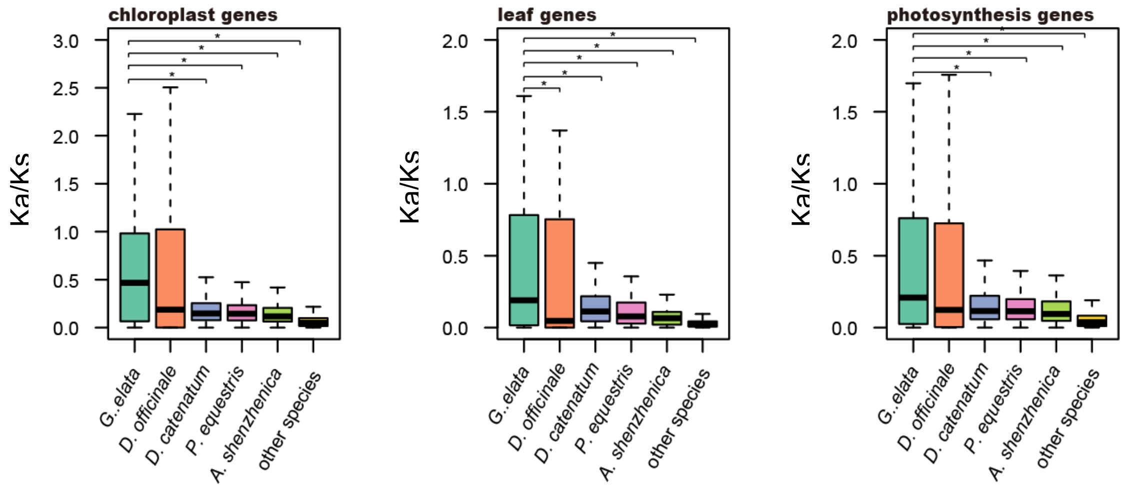

Supplementary Figure 4: Ka/Ks calculation in 3 nuclear-encoded photosynthesis-related gene sets.
Including 4,818 photosynthetic plastid genes, 203 leaf-development-related genes and 1,408 photosynthesis-related genes. **a)** 5ω test models calculating Ka/Ks in 3 nuclear-encoded photosynthesis-related gene sets. **b)** 6ω test models calculating Ka/Ks in 3 nuclear-encoded photosynthesis-related gene sets.

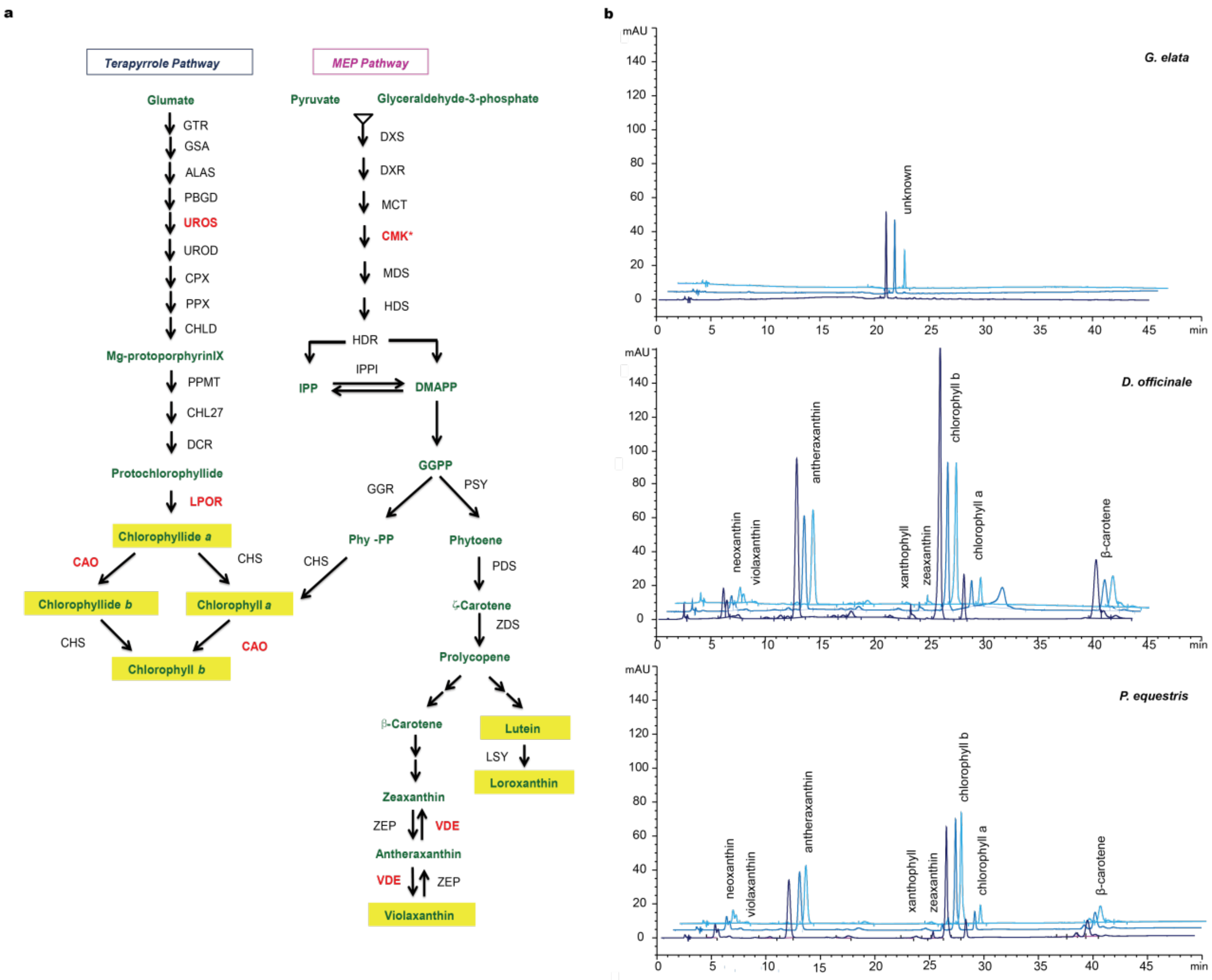

Supplementary Figure 5: Photosynthesis pigments synthesis pathway and HPLC detection.

a) From the molecular level, key enzymes lost in the genome are indicated by red, * indicates non-expression. b) From the quantitative way, using three different individual plant as parallel samples, using other two orchid plants as control group, we have not detected any photosynthesis pigment in *G. elata* plants.

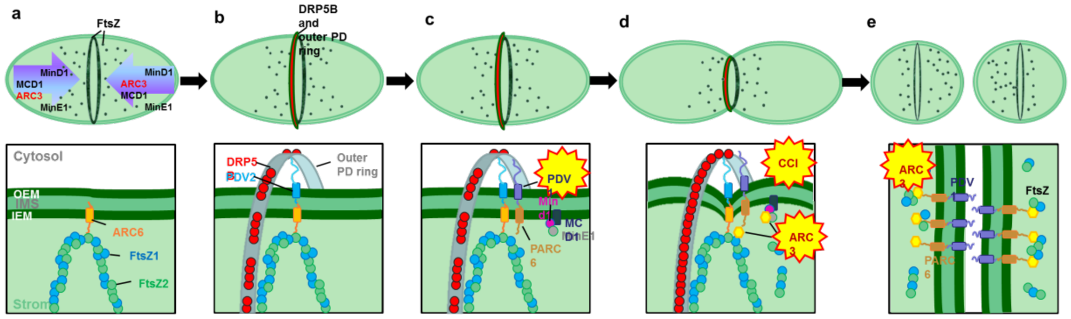

Supplementary Figure 6: The gene loss in the process of plastid division.
PDV1 and its interacting protein PDV2 regulate the rate of photosynthetic plant chloroplast division. PDV1 recruits the dynamin-related fission protein DRP5B and promotes DRP5B contractile activity with PDV2. ARC3, as part of the Min system, inhibits the Z-ring assembly at the non-division sites through direct interaction with FtsZ1, FtsZ2, and GCL, thus acting as a positive regulator of chloroplast division.
